# A bibliometric analysis of IL-35 research from 2009 to 2018

**DOI:** 10.7717/peerj.7992

**Published:** 2019-10-30

**Authors:** Xulong Cai, Chenrong Zhou, Li Zhou, Qiaolan Xu

**Affiliations:** Department of Pediatrics, Yancheng Third People’s Hospital, Yancheng, Jiangsu, China

**Keywords:** IL-35, Bibliometric analysis, CiteSpace, Infectious tolerance, Autoimmune, Central nervous system

## Abstract

**Background:**

Interleukin-35 (IL-35) is a recently discovered cytokine that plays a role in immune suppression and has therefore been the subject of a great deal of research. A bibliometric analysis of the global research concerning IL-35, however, is rare.

**Objectives:**

The aim of this research was to assess the international scientific output of IL-35 research and explore its hotspots and frontiers from 2009 to 2018 by bibliometric analysis.

**Methods:**

Publications about IL-35 research from 2009 to 2018 were retrieved from the Web of Science Core Collection (WoSCC). Citespace V was used to analyze years, journals, countries, research institutions, areas of exploration, research hotspots, and trends of publication.

**Results:**

We retrieved a total of 416 publications and observed a trend of publications increasing over the past decade. Original articles (351) were the most frequently occurring document type. The largest number of publications belonging to one country and one institution, respectively, was China (202) and Tianjin Medical University (17). Trending keywords may indicate frontier topics, including “infectious tolerance,” “autoimmune,” and “central nervous system.”

**Conclusion:**

This study provides valuable information on the study of IL-35 so that researchers may identify new research fields.

## Introduction

In 1997, a study reported that the Epstein-Barr Virus-Induced Gene 3 Protein (EBI3) associated noncovalently with the p35 subunit of interleukin −12 to form a heterodimeric hematopoietin in vivo (Devergne, Birkenbach & Kieff, 1997). The EBI3-p35 heterodimer has been named IL-35 by the International Union of Immunological Societies (IUIS) Subcommittee. The cytokine IL-35 has been identified as a member of the IL-12 family. Other IL-12 family cytokines include IL-12, IL-23, and IL-27.

IL-12 and IL-23 are both considered pro-inflammatory cytokines (Devergne, Birkenbach & Kieff, 1997). As an effective T cell immunomodulator, IL-27 has anti-inflammatory and pro-inflammatory properties (Thompson & Orr, 2018). Interleukin-35, however, was identified as an inhibitory cytokine in 2007 (Collison et al., 2007), indicating that IL-35 is quite different from other members of IL-12 family cytokines.

IL-35 is secreted by regulatory T cells and B cells (Collison et al., 2007; Shen et al., 2014). It has been shown that IL-35 inhibits the proliferation of T cells and induces the conversion of naïve T cells into iTr35 cells (Collison et al., 2007; Collison et al., 2010). IL-35 activated STAT1/STAT3 by means of an IL-35 receptor, and induced human B cells to transform into regulatory B-cells (Wang et al., 2014). After proinflammatory cytokines (tumor necrosis factor-a, interferon-γ, and IL-1β) provoke inflammation, IL-35 can be upregulated in human non-T cells, such as intestinal microvascular endothelial cells, primary aortic smooth muscle cells, and intestinal epithelial cell (Li et al., 2012). Studies have suggested that IL-35 is involved in autoimmune diseases, tumor progression, type II immune response, and infectious tolerance (Wang et al., 2014; Olson, Sullivan & Burlingham, 2013; Su et al., 2018; Shamji et al., 2019; Chatrabnous et al., 2019).

Interest in research on IL-35 has increased dramatically in recent years, and as a result, many journals have published articles on IL-35. The rapid growth of IL-35 literature renders identifying new developments and emerging trends in IL-35 research difficult. Few attempts, however, have been made to systematically analyze the knowledge, intellectual turning points, and key points in this field.

CiteSpace is a tool for visualizing and analyzing trends and patterns in scientific papers (Chen, 2004). CiteSpace provides a variety of functions to help understand network and historical patterns, including identifying rapidly growing subject areas, finding citation hotspots, decomposing networks into clusters, and automatically labeling clusters with citation terms (Chen, 2006). Here, we used bibliometric analysis to qualitatively and quantitatively evaluate IL-35 studies from 2009 to 2018. It is expected, therefore, that CiteSpace could be used to identify the emerging trends and hotspots of IL-35 research.

## Methods

All of the data obtained from the Web of Science Core Collection (WoSCC) of Thomson Reuters on February 27, 2019 was used in this study. The data retrieval strategy was: topic: (Interleukin-35) OR topic: (IL-35), index = SCI-EXPANDED, time span = 2009–2018 (retrieved date February 27, 2019). The following search string was used: document type: (Article OR Review). Web of Science database was used to analyze the characteristics of the literature, including the countries or regions in which it was conducted, the organization that researched IL-35, the journals it was published, as well as the research areas. The downloaded document records were exported to CiteSpace V for the further analysis.

## Results

### General information

**Figure 1:**
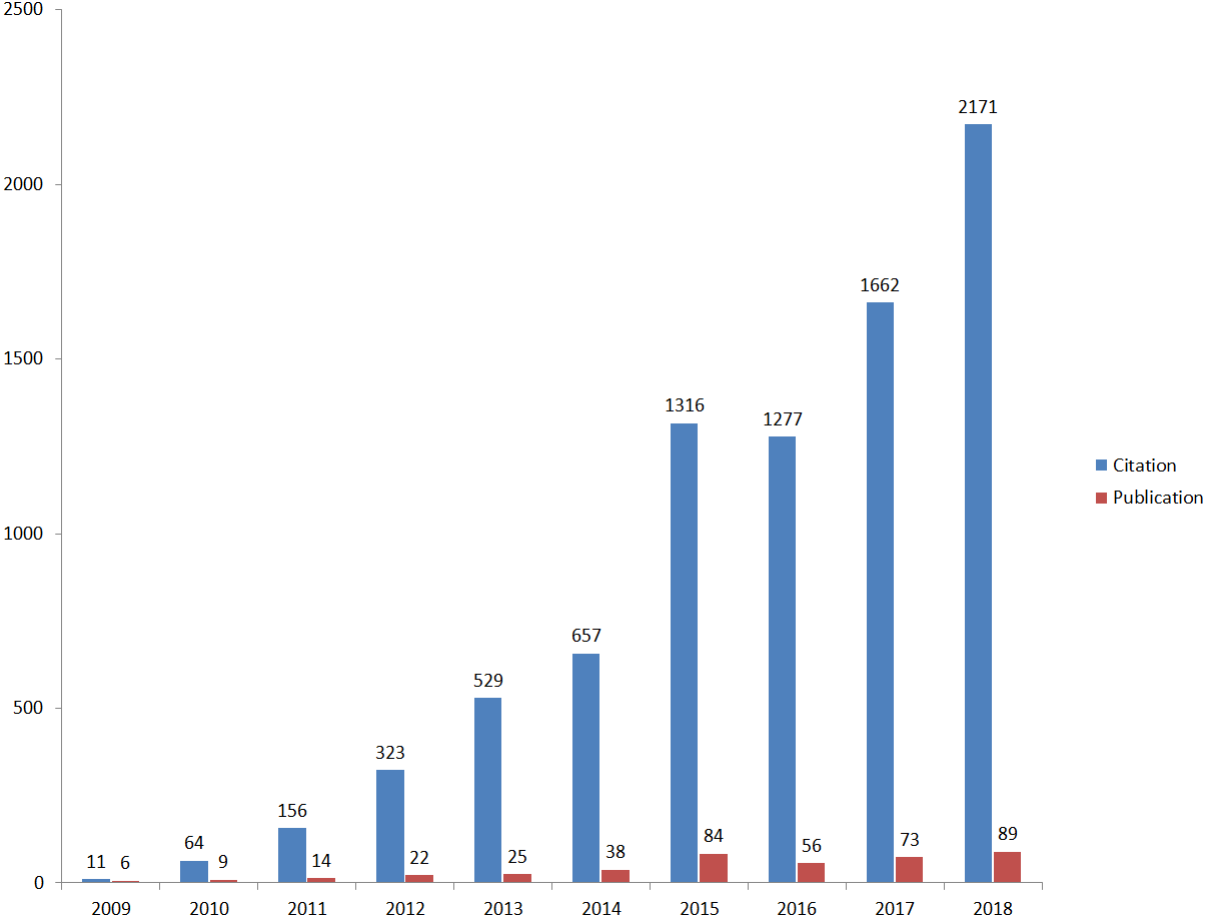
The number of publications and citations from 2009 to 2018. (A) The number of research papers published on il-35 was increasing from 2009 to 2018. (B) The frequency of citation of research papers on il-35 had increased significantly in the past decade.

We collected 416 papers on IL-35 the WoSCC. Of these, 351 (84.38%) were original articles. The number of published research papers increased during the years from 2009 (*n* = 6) to 2018 (*n* = 89). The number of citations of these papers also increased dramatically from 2009 (*n* = 11) to 2018 (*n* = 2, 171) (Fig. 1), reaching a total of 8166. There were 350 (84.13%) papers that had been cited at least once. Table 1 shows the 10 most frequently cited publications.

### Journal analysis

A total of 191 different journals published these articles. The maximum number of papers published in Cytokine was 19, followed by PLoS One (*n* = 16), the Journal of Immunology (*n* = 14), and Frontiers in Immunology (*n* = 10) (Table 2). These publications were cited by 1326 journals. Frontiers in Immunology had the largest number of citations (*n* = 223), followed by PLoS One (*n* = 167), the Journal of Immunology (*n* = 136) and Scientific Reports (*n* = 82).

### Country and institution analysis

Research on IL-35 was found to have been conducted in 41 countries. China was the country with the largest number of published papers (*n* = 202), followed by the USA (*n* = 91), and Germany (*n* = 26). Over 500 research institutions had published 416 articles on IL-35. 118 papers had been published by the top 10 research institutes. The most publications originated from Tianjin Medical University (*n* = 17), followed by St. Jude Children’s Research Hospital (*n* = 15), and the National Institutes of Health NIH USA (*n* = 14). (Tables 3–4)

**Table 1:** Top 10 most cited articles on IL-35.

Rank	First Author	Year	Title	Journal	Impact Factor (2017)	Cited
1	Lauren W. Collison	2010	IL-35-mediated induction of a potent regulatory T cell population	Nature Immunology	21.8	408
2	Ping Shen	2014	IL-35-producing B cells are critical regulators of immunity during autoimmune and infectious diseases	Nature	41.6	378
3	Ren-Xi Wang	2014	Interleukin-35 induces regulatory B cells that suppress autoimmune disease	Nature Medicine	32.6	254
4	Lauren W. Collison	2012	The composition and signaling of the IL-35 receptor are unconventional	Nature Immunology	21.8	181
5	Lauren W. Collison	2009	Regulatory T Cell Suppression Is Potentiated by Target T Cells in a Cell Contact, IL-35-and IL-10-Dependent Manner	The Journal of Immunology	4.54	150
6	Veronika Bachanova	2014	Clearance of acute myeloid leukemia by haploidentical natural killer cells is improved using IL-2 diphtheria toxin fusion protein	Blood	15.1	149
7	Irina Kochetkova	2010	IL-35 Stimulation of CD39(+) Regulatory T Cells Confers Protection against Collagen II-Induced Arthritis via the Production of IL-10	The Journal of Immunology	4.54	133
8	Xinyuan Li	2012	IL-35 Is a Novel Responsive Anti-inflammatory Cytokine - A New System of Categorizing Anti-inflammatory Cytokines	PLoS One	2.77	110
9	Gregory S. Whitehead	2012	IL-35 production by inducible costimulator (ICOS)-positive regulatory T cells reverses established IL-17-dependent allergic airways disease	Journal of Allergy and Clinical Immunology	13.3	110
10	Stefan Wirtz	2011	Interleukin-35 Mediates Mucosal Immune Responses That Protect Against T-Cell-Dependent Colitis	Gastroenterology	20.8	106

### Research area analysis

**Figure 2:**
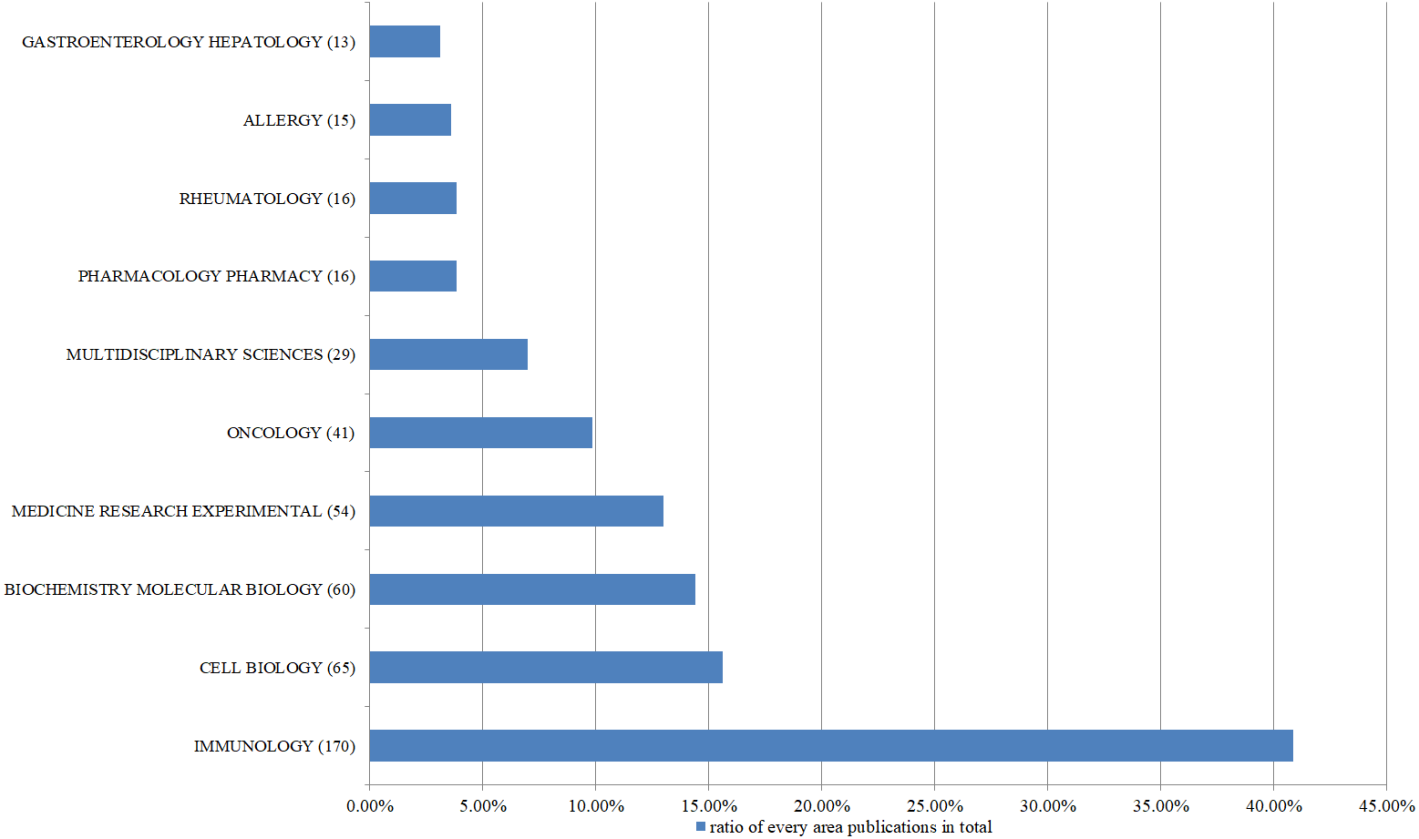
The top 10 most frequently appearing research areas in IL-35 studies from 2009 to 2018. In recent 10 years, the research on IL-35 mainly involves immunology.

A total of 50 research field were represented, with the majority of publications focusing on immunology (*n* = 171), cell biology (*n* = 65), and biochemistry-molecular biology (*n* = 60). Figure 2 shows the top 10 research fields in IL-35 papers from 2009 to 2018.

**Table 2:** The top 5 most productive and cited journals.

Rank	Productive journals	The number of published papers	Rank	Cited journal	Cited frequency
1	Cytokine	19	1	Frontiers in Immunology	223
2	PLoS One	16	2	PLoS One	167
3	The Journal of Immunology	14	3	The Journal of Immunology	136
4	Frontiers in Immunology	10	4	Scientific Reports	82
5	Journal of Interferon and Cytokine Research	8	5	Cytokine	75

### Co-citation analysis

**Figure 3:**
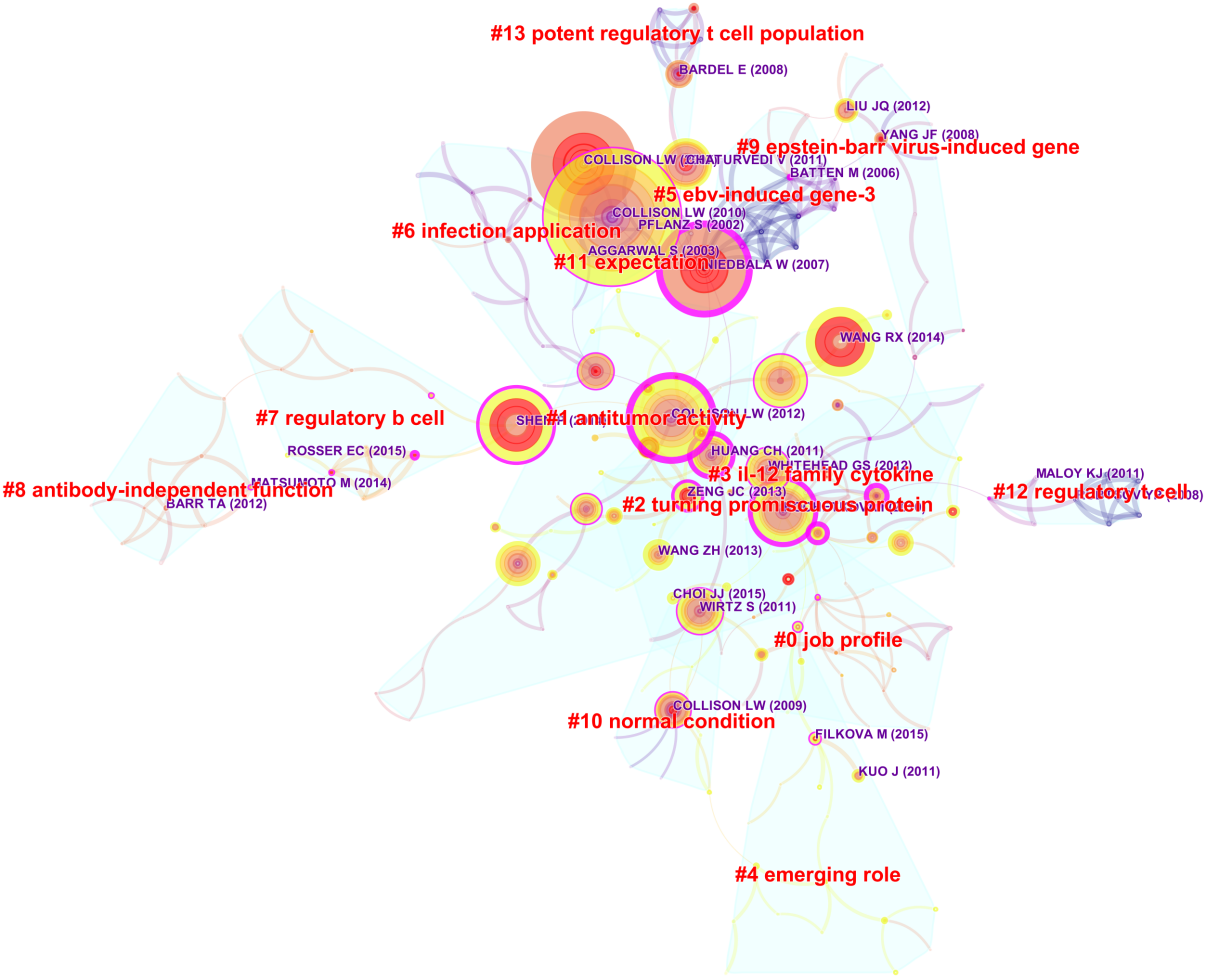
Reference co-citation map of articles related to IL-35 research published from 2009 to 2018. (A) Red labels represented different clusters. (B) “#0, #1, #2, #3…” were the serial number of the clusters. (C) The red circle of the node indicated that the citation frequency of the literature increases suddenly in a given period of time.

**Figure 4:**
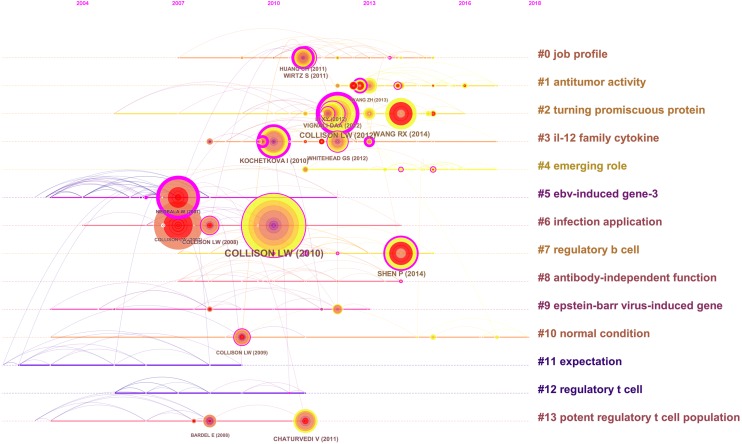
Reference co-citation time-view map of articles related to IL-35 research published from 2009 to 2018. (A) From #0 to #13, the clusters size decreased gradually. (B) From left to right, it means the time span from the past to 2018.

The map analysis was shown by a literature co-citation network. The network contains 225 nodes and 446 links. The Modularity Q was 0.8258 (>0.5), meaning that the clusters of networks were reasonable. The Mean Silhouette was 0.5107, indicating that the homogeneity of clusters was, on average, acceptable. As shown in Fig. 3, nodes represent referenced documents. The largest cluster in the visualization is #0 job profile, followed by #1 antitumor activity, #2 turning promiscuous protein, and #3 IL-12 family cytokine. These clusters were also shown in a timeline view (Fig. 4).

**Table 3:** Top 10 prolifc countries publishing paper on IL-35.

Rank	Country	Frequency
1	China	202
2	USA	91
3	Germany	26
4	Iran	18
5	Japan	16
6	Italy	11
7	Turkey	10
8	Canada	9
9	England	8
10	France	8

**Table 4:** Top 10 prolifc institutions publishing paper on IL-35.

Rank	Institution	Frequency
1	TIANJIN MEDICAL UNIVERSITY	17
2	ST JUDE CHILDREN S RESEARCH HOSPITAL	15
3	NATIONAL INSTITUTES OF HEALTH NIH USA PENNSYLVANIA COMMONWEALTH SYSTEM OF HIGHER EDUCATION	14
4	PCSHE	14
5	SHANDONG UNIVERSITY	13
6	ANHUI MEDICAL UNIVERSITY	9
7	CHINA MEDICAL UNIVERSITY	9
8	HUAZHONG UNIVERSITY OF SCIENCE TECHNOLOGY	9
9	JILIN UNIVERSITY	9
10	NIH NATIONAL EYE INSTITUTE NEI	9

### Keywords analysis

**Figure 5:**
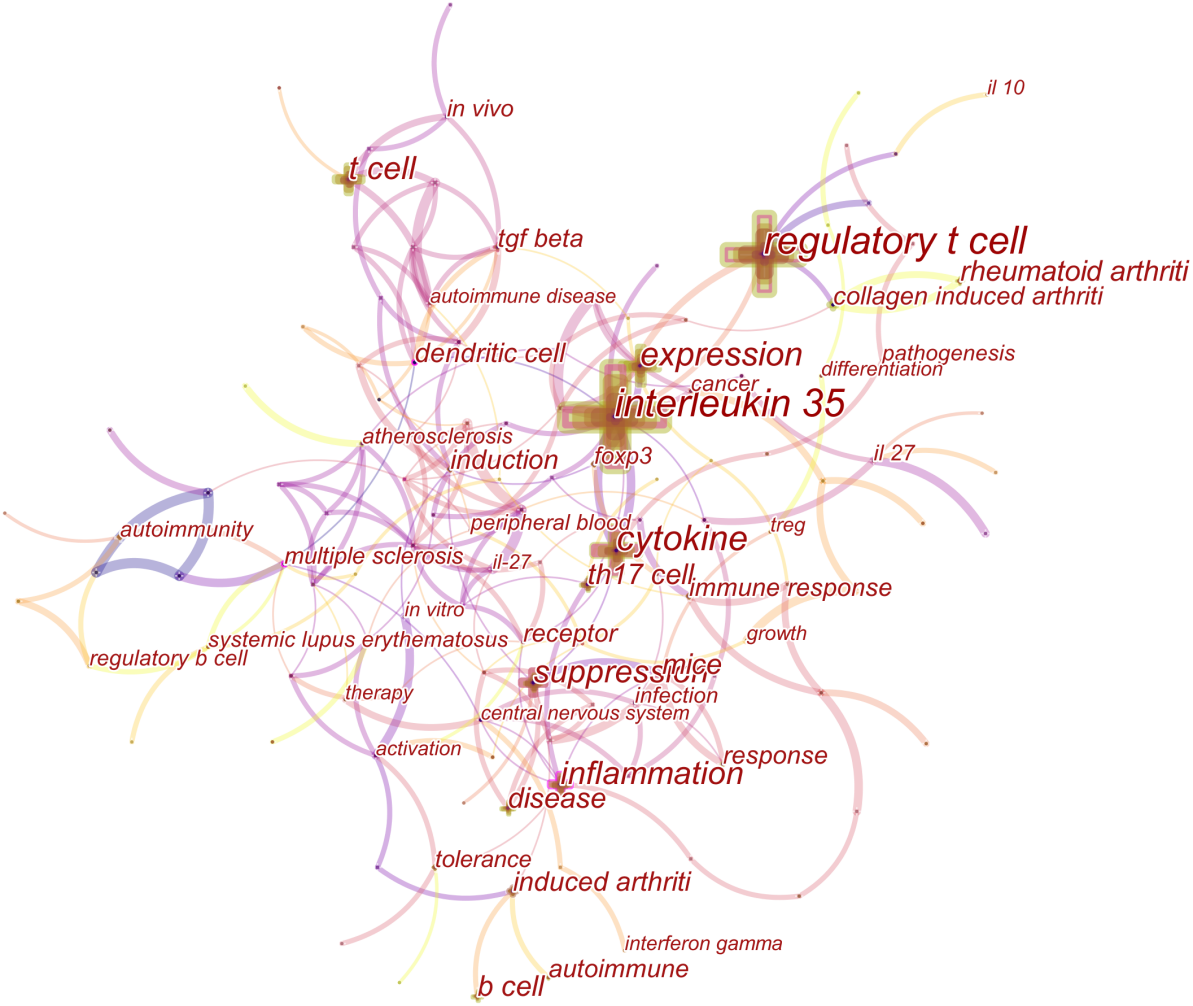
A keyword co-occurrence map of IL-35 from 2009 to 2018. The node size represented the co-occurrence frequency of keyword. The larger the node size indicated the higher the keyword frequency.

**Figure 6:**
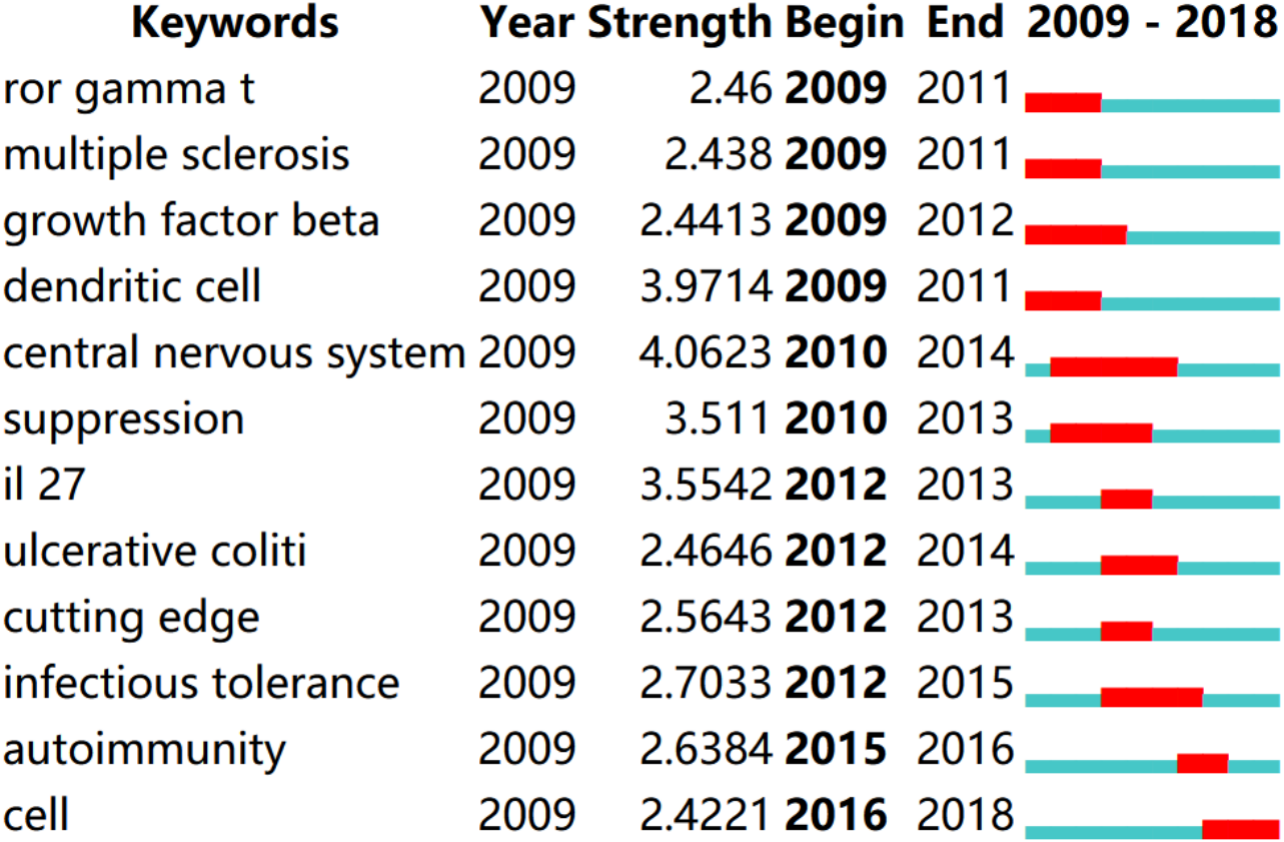
Top 12 keywords with strongest citation bursts. Burst strength: the intensity of the sudden increase of keyword citation frequency in a certain period of time. Research frontiers are identified based on burst keywords.

We used CiteSpaceV to analyze keywords. Over a period of time, a knowledge map of the cooccurrence of keywords may reflect hot topics, whereas trending keywords may indicate frontier topics. Generating a visual knowledge map of keyword cooccurrence resulted in 130 nodes and 431 links (Fig. 5). The strongest citation bursts keywords were as follows: “central nervous system,” “dendritic cell,” and “IL-27” (Fig. 6).

## Discussion

In this study, we analyzed the structure of the citation network and the trends in topics of recent research involving IL-35. An analysis of the literature published about IL-35 over the past 10 years showed a high growth rate of publications related to IL-35 with frequent citations, indicating that the study of IL-35 was a hot topic.

The top 10 institutions that were engaged in IL-35 research contributed to 118 publications, accounting for 28.37% of the total number of research papers. Of the top ten, six institutions were from China. Over the past 10 years, China has been the leading country in IL-35 research, indicating China’s great progress in the life sciences.

In order to compare the reception of each publication, we also analyzed the citation frequency of the papers. The results showed that an article published in Nature Immunology (Collison et al., 2010) was the most frequently cited article, indicating that “IL-35-mediated induction of a potent regulatory T cell population” was an important publication to reference in IL-35 research.

The co-citation knowledge map refers to a network of co-citation publications to determine research frontiers. These nodes represent different documents, marked by the year of publication and the author of the publication. The size of the node is proportional to the number of references cited in specific time periods. The red citation ring indicates a sudden increase of citations over a period of time. Trending citations provide an effective way to track research hotspots. Furthermore, we analyzed the characteristics related to clusters of references and constructed a visual map of this research that contained 225 nodes and 446 links. The color of the node indicates how recently the relevant literature had been published. The dark color corresponds to older research, the yellow or orange color to new research (Chen et al., 2012). Among the 14 clusters, Cluster 0 (job profile) is the largest. The topics of Cluster 1 (antitumor activity), Cluster 2 (turning promiscuous protein), Cluster 4 (emerging role), and Cluster 7 (regulatory B cell) were the newest research . The publications that comprised Cluster 1 (antitumor activity) focused on the role of IL-35 in cancer, including the severity of the malignancy, the clinical stage of the tumor, the promoting of tumor growth, the autocrine growth factor, and the limited antitumor immunity (Zeng et al., 2013; Wang et al., 2013; Nicholl et al., 2014; Turnis et al., 2016).

Cluster 2 (turning promiscuous protein) publications focused on unconventional proteins, including unconventional modes of signaling, and the suppression of autoimmune disease and responsive anti-inflammatory cytokines (Li et al., 2012; Collison et al., 2012; Wang et al., 2014).

The cluster 4 (emerging role) research reported that the low serum level of IL-35 is related to both active systemic lupus erythematosus and active rheumatoid arthritis (Li et al., 2012; Collison et al., 2012; Wang et al., 2014). Interestingly, some studies reported that IL-35 promotes chronic inflammation (Filková et al., 2015; Thiolat et al., 2014).

The publications corresponding to cluster 7 (regulatory B cell) focused on the function of the regulatory B cell, including inflammation, autoimmunity, and the maintenance of the fine equilibrium required for infectious tolerance (Iwata et al., 2011; Yoshizaki et al., 2012; Mauri & Bosma, 2012; Flores-Borja et al., 2013).

Bursts of keywords provide a reasonable forecasting of the research frontier. Citespace detected several bursts which were regarded as an indicator of the frontiers of IL-35 research. In the Fig. 6 , the blue line represents the time interval. The start to the end of each burst interval is indicated by a red line. Therefore, the top three research frontiers of IL-35 were as follows:

(1) “infectious tolerance”: Infection tolerance is an in vivo process in which tolerance is transferred from one group of lymphocytes to another. In this way, short-term treatment aimed at producing infection tolerance may lead to long-term, self-sustaining immune homeostasis in clinical settings (Kendal & Waldmann, 2010). IL-35 plays an important role in infection tolerance (Olson, Sullivan & Burlingham, 2013; Tao et al., 2015).

(2) “autoimmunity”: Studies have found abnormal expression of IL-35 in patients suffering from autoimmune diseases, including rheumatoid arthritis, systemic lupus erythematosus, inflammatory bowel disease, multiple sclerosis, diabetes mellitus type 1, psoriasis, autoimmune hepatitis, multiple sclerosis, and experimental autoimmune uveitis (Choi et al., 2015; Su et al., 2018). An autoimmune disease is a pathophysiological state; the immune response directly targets and damages the body’s own tissues.

(3) “central nervous system”: Through the study of a mouse model, IL-35 was found to be associated with central nervous system demyelination, autoimmune encephalomyelitis, and the control of host responses following central nervous system viral infection (Zandian et al., 2011; Tirotta et al., 2013; Xie et al., 2016). A study suggested that the level of IL-35 in patients with neuromyelitis optica spectrum disorders was low. This might be an important biomarker of the severity of neuromyelitis optica spectrum disorders (Zhang et al., 2016).

## Conclusions

This study will help researchers understand the trends of IL-35 research. Infectious tolerance and autoimmune diseases may be the latest research frontiers. The molecular biological mechanisms of IL-35 need further exploration.

##  Supplemental Information

10.7717/peerj.7992/supp-1 Supplemental Information 1 Raw data

## Additional Information and Declarations

### Data Availability

The following information was supplied regarding data availability: The raw data is available in the Supplemental File.
